# Assessment of global myocardial perfusion reserve using cardiovascular magnetic resonance of coronary sinus flow at 3 Tesla

**DOI:** 10.1186/1532-429X-16-24

**Published:** 2014-03-27

**Authors:** Vineet K Dandekar, Michael A Bauml, Andrew W Ertel, Carolyn Dickens, Rosalia C Gonzalez, Afshin Farzaneh-Far

**Affiliations:** 1 Section of Cardiology, Department of Medicine, University of Illinois at Chicago, 840 South Wood St. M/C 715, Suite 920 S, Chicago, IL 60612, USA; 2 National Heart, Lung, and Blood Institute, National Institutes of Health, Bethesda MD, USA; 3 Department of Radiology, University of Illinois at Chicago, Chicago, IL, USA; 4 Division of Cardiology, Department of Medicine, Duke University, Durham, NC, USA

**Keywords:** Cardiovascular magnetic resonance, Stress testing, Perfusion, Regadenoson

## Abstract

**Background:**

Despite increasing clinical use, there is limited data regarding regadenoson in stress perfusion cardiovascular magnetic resonance (CMR). In particular, given its long half-life the optimal stress protocol remains unclear. Although Myocardial Perfusion Reserve (MPR) may provide additive prognostic information, current techniques for its measurement are cumbersome and challenging for routine clinical practice.

The aims of this study were: 1) To determine the feasibility of MPR quantification during regadenoson stress CMR by measurement of Coronary Sinus (CS) flow; and 2) to investigate the role of aminophylline reversal during regadenoson stress-CMR.

**Methods:**

117 consecutive patients with possible myocardial ischemia were prospectively enrolled. Perfusion imaging was performed at 1 minute and 15 minutes after administration of 0.4 mg regadenoson. A subgroup of 41 patients was given aminophylline (100 mg) after stress images were acquired. CS flow was measured using phase-contrast imaging at baseline (pre CS flow), and immediately after the stress (peak CS flow) and rest (post CS flow) perfusion images.

**Results:**

CS flow measurements were obtained in 92% of patients with no adverse events. MPR was significantly underestimated when calculated as peak CS flow/post CS flow as compared to peak CS flow/pre CS flow (2.43 ± 0.20 vs. 3.28 ± 0.32, p = 0.03). This difference was abolished when aminophylline was administered (3.35 ± 0.44 vs. 3.30 ± 0.52, p = 0.95). Impaired MPR (peak CS flow/pre CS flow <2) was associated with advanced age, diabetes, current smoking and higher Framingham risk score.

**Conclusions:**

Regadenoson stress CMR with MPR measurement from CS flow can be successfully performed in most patients. This measurement of MPR appears practical to perform in the clinical setting. Residual hyperemia is still present even 15 minutes after regadenoson administration, at the time of resting-perfusion acquisition, and is completely reversed by aminophylline. Our findings suggest *routine* aminophylline administration may be required when performing stress CMR with regadenoson.

## Background

Perfusion cardiovascular magnetic resonance (CMR) is increasingly used for diagnosis and risk stratification of patients with known or suspected coronary artery disease (CAD). The majority of these procedures are performed using the non-selective vasodilator agent adenosine. Regadenoson is a relatively new selective adenosine A2A receptor agonist, and is now the most widely used stress agent in the United States [1]. Unlike adenosine, regadenoson is conveniently given as a single fixed-dose bolus rather than as a weight adjusted infusion and is associated with fewer side-effects [2,3]. However, in contrast to Single-Photon Emission Computed Tomography (SPECT) imaging [1-3], there is limited data on regadenoson use in CMR [4-8]. In particular, due to regadenoson’s longer and multi-exponential clearance [9], a major unresolved issue is the impact of this on the typical stress-rest CMR stress protocol. In some centers, reversal is facilitated with aminophylline before resting images are acquired. However, there is significant variation in current practice and the role of routine aminophylline reversal is unclear.

Myocardial perfusion reserve (MPR) is the ratio of global myocardial blood flow at stress vs. rest. Traditionally, MPR has been measured non-invasively using quantitative positron emission tomography (PET) or CMR [10-12]. Several recent studies have suggested that measurement of MPR provides significant additive prognostic information during stress perfusion imaging in patients with known or suspected CAD [13-16]. However, both PET and current CMR techniques are cumbersome, and in the case of PET require radiation as well as on-site Rubidium-82 generators. Therefore, they are challenging for routine clinical practice and have been limited to specialized research centers. An alternative, simple CMR method for measurement of MPR by quantifying change in coronary sinus (CS) flow has been described [17,18]. The CS drains approximately 96% of total myocardial blood flow and provides a potentially convenient location for measurement of global myocardial blood flow [19]. This method has been validated against both invasive and PET techniques [18,20]. We hypothesized that measurement of CS flow at stress and rest may provide a simple and rapid assessment of MPR during regadenoson stress perfusion CMR.

The aims of this study were therefore two-fold: 1) To determine the feasibility of MPR quantification during regadenoson stress CMR by measurement of CS flow; and 2) to investigate the role of aminophylline reversal during regadenoson stress-CMR.

## Methods

### Study population

One hundred and seventeen consecutive patients with suspected myocardial ischemia referred for CMR stress testing were prospectively enrolled in a single academic medical center between March 2012 and April 2013. Patients were excluded if they had metallic implants incompatible with CMR, glomerular filtration rate < 30 ml/min, high degree atrio-ventricular block, severe active wheezing from asthma or severe claustrophobia. Subjects were asked to abstain from caffeine-containing products for at least 12 hours prior to the test. Information on baseline demographic variables and prior laboratory testing was obtained from patient interviews and the electronic medical record. Patients gave informed written consent for the protocol, which was approved by the University of Illinois Institutional Review Board.

### CMR

**Figure 1:**
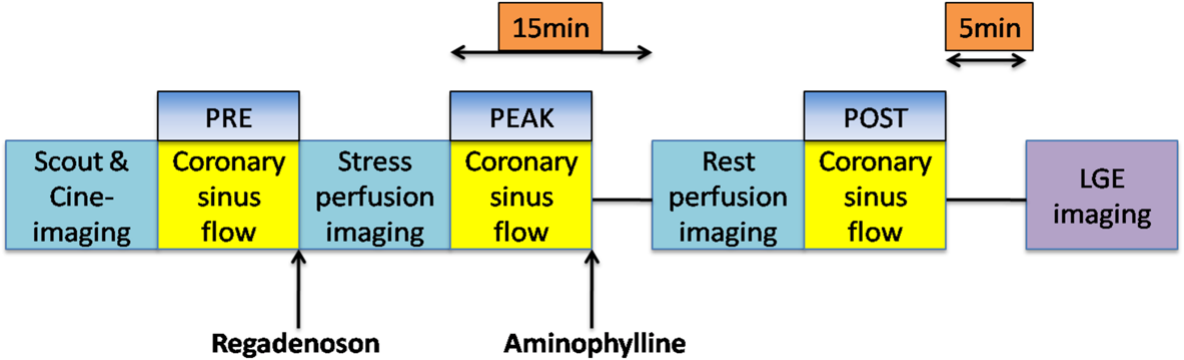
**Regadenoson CMR stress protocol.** Stress perfusion protocol showing timing of administration of regadenoson and aminophylline. Coronary sinus flow was measured using phase-contrast imaging at three time-points (pre-stress, peak stress, and post stress). LGE = late gadolinium enhancement.

Images were acquired on a 3 T scanner (Philips Achieva, Philips Medical Systems, Best, the Netherlands) using a six-element phased-array receiver coil. Steady-state free-precession cine images were acquired in multiple short-axis and three long-axis views (repetition time 3.0 ms; echo time 1.5 ms; flip angle 40°; slice thickness 6 mm) (Figure 1).

The patient table was then partially pulled outside the scanner bore to allow direct observation of the patient and full access. A 0.4 mg bolus of regadenoson (Lexiscan, Astellas Pharma Inc) was infused under continuous electrocardiography and blood pressure monitoring. Approximately 1 minute after regadenoson administration, the perfusion sequence was applied and Gadolinium contrast (0.075 mmol/kg gadoteridol, Bracco Diagnostics) followed by a saline flush (30 ml) was infused (4.5 ml/s) via an antecubital vein. On the console, the perfusion images were observed as they were acquired, with breath-holding starting from the appearance of contrast in the right ventricular cavity. Imaging was completed 10 to 15 s after the gadolinium bolus had transited the left ventricular myocardium. Perfusion images consisted of three to four short-axis slices obtained every heartbeat with a saturation-recovery, gradient-echo sequence (repetition time 2.8 ms; echo time 1.1 ms; flip angle 20°; voxel size 2.5 × 2.5 × 8 mm). A random subgroup of 41 patients was administered aminophylline 100 mg IV immediately after stress perfusion imaging. Rest perfusion images were acquired 15 minutes after stress imaging with an additional contrast bolus (0.075 mmol/kg gadoteridol) using identical sequence parameters. Five minutes after rest perfusion, late gadolinium enhancement (LGE) imaging was performed with a 2D segmented gradient echo phase-sensitive inversion-recovery sequence in the identical views as cine-CMR. Inversion delay times were typically 280 to 360 ms (Figure 1).

### Coronary sinus imaging

**Figure 2:**
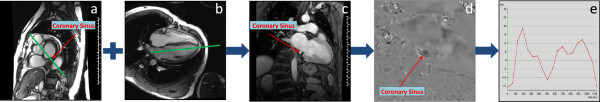
**Coronary sinus flow measurement.** The coronary sinus was identified in the atrio-ventricular groove, on the basal slices of the short-axis stack (panel** a**). the plane for flow measurement was prescribed parallel to the long-axis of the heart on the 4-chamber view and perpendicular to the direction of flow in the coronary sinus, approximately 0.5 cm from the ostium (green line, panels **a** and **b**). the proximal coronary sinus was seen in cross-section on the phase contrast images (panels **c** and **d**). flow vs time curves were generated by drawing a region of interest around the coronary sinus to calculate through-plane flow (panel **e**).

The coronary sinus was identified in the atrio-ventricular groove, on the basal slices of the short-axis stack (Figure 2). The plane for flow measurement was prescribed parallel to the long-axis of the heart on the 4-chamber view and perpendicular to the direction of flow in the coronary sinus, approximately 0.5 cm from the ostium (green line Figure 2). Velocity-encoded images were acquired with retrospective ECG gating during 12- to 18-second breath holds (slice thickness 6 mm; in-plane resolution 1.3 × 1.3 mm; temporal resolution 35–55 ms; and velocity encoding 60 cm/s). These images were acquired in the baseline (pre) state as well as immediately after stress (peak) and rest (post) perfusion images (Figure 1). In a random subgroup of 50 patients the acquisition of the baseline (pre) and stress (peak) CS images was performed again for assessment of repeatability.

### CMR analysis

Perfusion and LGE images were visually interpreted by standard methods [21]. For perfusion-CMR, stress and rest images were read side-by-side. Blinded quantitative analysis of CS flow was performed using commercial software (Philips View Forum, Best, the Netherlands). The contour of the coronary sinus was traced on the phase-contrast magnitude images throughout the cardiac cycle (Figure 2). Coronary sinus flow was calculated by integrating the momentary flow rate values from each cardiac phase over the entire cardiac cycle and multiplying by the mean heart rate during the acquisition (Figure 2). Coronary sinus flow was calculated at baseline (pre), immediately after the stress (peak) and at rest (post). A second blinded physician analyzed the second set of baseline (pre) and stress (peak) images obtained in the randomly selected group of 50 patients.

### Statistical analysis

Normally distributed data were expressed as mean ± SD. Interobserver repeatability was analyzed using the Bland-Altman method [22]. Continuous variables were compared by the Student’s t-test or Wilcoxon rank-sum (depending on data normality). Comparisons of discrete variables were made using the chi-square test; Fisher’s exact test was used when the assumptions of the chi-square test were not met. A p-value of <0.05 was considered statistically significant.

## Results

### Study population

**Figure 3:**
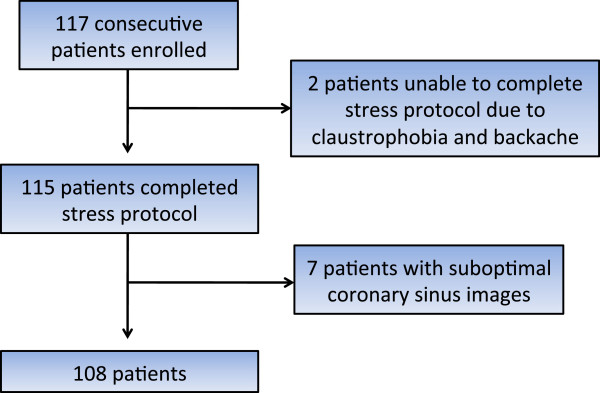
Study enrollment and completion.

Patients were enrolled at the time of presentation for the clinically ordered stress CMR scan. All patients gave informed consent. Out of this initial enrolled population of 117 patients, two patients were excluded due to severe claustrophobia. One of these patients also had severe backache. No patients were seen (or excluded) with active bronchospasm, high-grade AV block, metallic implants incompatible with CMR, or glomerular filtration rate < 30 ml/min. Seven patients had inadequate image quality of the CS. The main causes of poor image quality were inability to breath-hold adequately, poor gating, patient movement, and inability to identify the coronary sinus. Therefore the full protocol was successfully completed in 108 patients (92%) (Figure 3).

### Patient characteristics

Table 1 summarizes the baseline patient characteristics. The mean age of the study population was 60 ± 14 yrs. Forty-six percent of patients were male and 31% had diabetes mellitus. Thirty-six percent had known coronary artery disease, and 19% were current smokers. The mean ejection fraction was 65 ± 12%. The mean 10-year Framingham risk score was 23 ± 18.

**Table 1:** Baseline characteristics

**Characteristic**	**Value**
	**(n = 108)**
**Age ± SD**	60 ± 14
**Male sex (%)**	50 (46)
**Caucasian (%)**	20 (19)
**Diabetes (%)**	34 (31)
**Hypertension (%)**	79 (73)
**Hyperlipidemia (%)**	58 (54)
**BMI ± SD**	31 ± 6
**Known CAD (%)**	39 (36)
**Family history of CAD (%)**	24 (22)
**Current smoker (%)**	21 (19)
**Former smoker (%)**	24 (22)
**Asthma/COPD (%)**	6 (5)
**Ejection fraction ± SD**	65 ± 12
**10 yr Framingham risk ± SD**	23 ± 18

BMI = body/mass index; CAD = coronary artery disease, COPD = Chronic Obstructive Pulmonary Disease, GFR = glomerular filtration rate; SD = standard deviation.

### Safety and side effects

Forty-nine percent of patients experienced at least one side effect after administration of regadenoson (Table 2). The most commonly reported symptom was shortness of breath (24%), followed by chest pain (18%), headache (14%), flushing (13%), nausea (12%), and dizziness (8%). There were no life-threatening adverse events and no instances of atrio-ventricular block or bronchospasm. In addition there was no incidence of hypotension, contrast reactions, heart failure events, unstable angina or patients requiring antianginal treatment.

**Table 2:** Side-effects of regadenoson during stress CMR

**Symptom**	**Frequency**
**Dyspnea**	24%
**Chest pain**	18%
**Headache**	14%
**Flushing**	13%
**Nausea**	12%
**Dizziness**	8%
**Any symptom**	49%

### Coronary sinus flow measurements

Acquisition of CS images added approximately 2–3 minutes to overall scanning time with an additional 5 minutes required for off-line quantitative flow analysis. The flow profile obtained was typically biphasic with a first peak in early systole and a second peak during early diastole (Figure 2).

### Comparison of different myocardial perfusion reserve calculations

**Figure 4:**
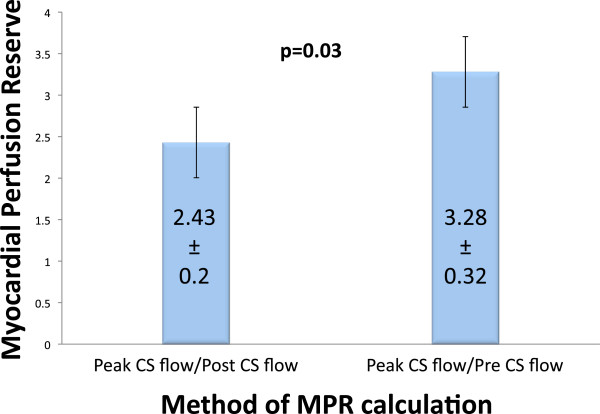
**Comparison of different MPR calculations.** MPR is significantly greater when using peak CS flow/pre CS flow compared with peak CS flow/post CS flow.

MPR was calculated in two different ways: 1) peak CS flow/pre CS flow, and 2) peak CS flow/post CS flow (Figure 1). In the entire study population, MPR calculated as peak CS flow/pre CS flow was significantly greater than when calculated as peak CS flow/post CS flow (3.28 ± 0.32 vs. 2.43 ± 0.20, p = 0.03) (Figure 4).

### Interobserver repeatability

**Figure 5:**
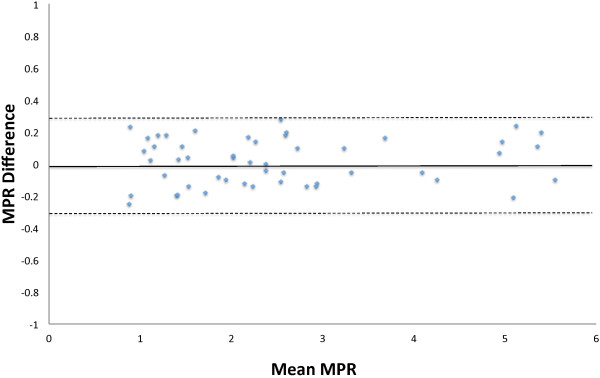
**Bland-Altman analysis of interobserver repeatability for MPR.** Solid line represents the bias. Dashed lines represent the limits of agreement.

Bland-Altman analysis of interobserver repeatability for MPR (calculated as peak CS flow/pre CS flow) showed a bias of −0.015 (CI −0.056 to 0.026). Limits of agreement were −0.303 to 0.273. The Bland-Altman plot showed no systematic bias (Figure 5).

### Effects of aminophylline

**Figure 6:**
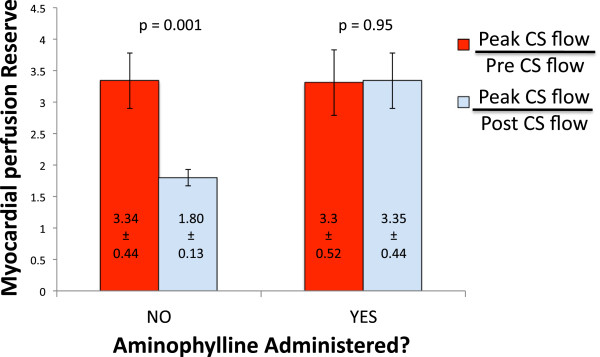
**Effect of aminophylline on MPR calculations.** In the subgroup of patients not receiving aminophylline, MPR measurement is significantly lower when using peak CS flow/post CS flow compared with peak CS flow/pre CS flow. In those receiving aminophylline, there is no difference in MPR between the two methods of calculation. CS = coronary sinus.

**Figure 7:**
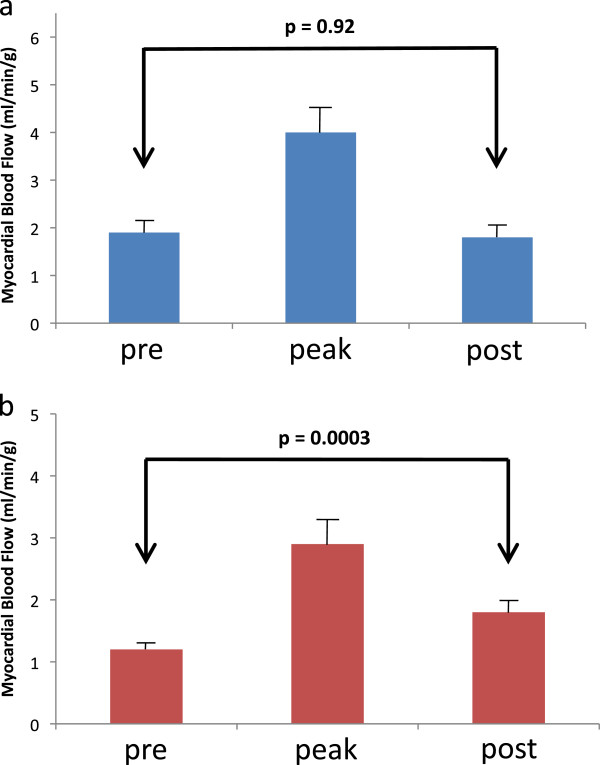
**Absolute coronary sinus flow during regadenoson stress protocol.** Absolute coronary sinus flow (±SEM) measured at three time-points (pre-stress, peak stress, post stress). In patients receiving aminophylline (panel **a**) and in patients not receiving aminophylline (panel **b**) demonstrates significant residual hyperemia in the absence of aminophylline.

Table 3 shows baseline patient characteristics of the patients receiving aminophylline vs those who did not. In the subgroup of patients receiving aminophylline, there was no significant difference between MPR calculated as peak CS flow/post CS flow vs. peak CS flow/pre CS flow (3.35 ± 0.44 vs. 3.30 ± 0.52, p = 0.95) (Figure 6). In contrast, among patients not receiving aminophylline, MPR calculated as peak CS flow/pre CS flow was significantly greater than MPR calculated as peak CS flow/post CS flow (3.34 ± 0.44 vs. 1.80 ± 0.52, p = 0.001) (Figure 6). Figure 7a shows that absolute post CS flow (in ml/min/g) returned to pre CS values when patients are administered aminophylline, suggesting complete reversal of residual hyperemia from regadenoson. In contrast, post CS flow remains elevated compared to pre CS flow in the absence of aminophylline presumably due to residual hyperemia from regadenoson (Figure 7b).

**Table 3:** Baseline characteristics of patients receiving aminophylline vs those not receiving Aminophylline

**Characteristic**	**Aminophylline**	**No Aminophylline**	**P Value**
	**(n = 41)**	**(n = 67)**	
**Age ± SD**	63 ± 17	59 ± 17	0.24
**Male sex (%)**	22 (54)	28 (42)	0.31
**Caucasian (%)**	9 (22)	11 (16)	0.54
**Diabetes (%)**	8 (20)	26 (39)	0.06
**Hypertension (%)**	31 (76)	48 (72)	0.90
**Hyperlipidemia (%)**	26 (63)	32 (48)	0.17
**Current smoker (%)**	8 (20)	13 (19)	0.93
**10 yr Framingham risk ± SD**	24 ± 14	23 ± 20	0.75
**Baseline heart rate**	71 ± 12	69 ± 15	0.47
**Baseline blood pressure:**			
**Systolic**	129 ± 19	124 ± 14	0.12
**Diastolic**	66 ± 15	68 ± 19	0.57
**Peak heart rate**	96 ± 17	93 ± 18	0.39
**Peak blood pressure:**			
**Systolic**	126 ± 18	125 ± 15	0.76
**Diastolic**	63 ± 15	67 ± 18	0.24

BMI = body/mass index; CAD = coronary artery disease; SD = standard deviation. MPR was calculated as peak CS flow/pre CS flow.

### Patient characteristics stratified by MPR

Table 4 shows baseline patient characteristics stratified by MPR < 2 vs MPR ≥ 2. MPR was calculated as peak CS flow/pre CS flow. Patients with MPR < 2 were significantly more likely to have advanced age, diabetes, higher Framingham risk score, and history of current smoking.

**Table 4:** Baseline characteristics stratified by myocardial perfusion reserve (MPR)

**Characteristic**	**MPR < 2 (n = 42)**	**MPR ≥ 2 (n = 66)**	**P value**
**Age ± SD**	64 ± 15	58 ± 14	0.04
**Male sex (%)**	23 (55)	27 (41)	0.16
**Caucasian (%)**	9 (21)	11 (17)	0.39
**Diabetes (%)**	18 (43)	16 (24)	0.04
**Hypertension (%)**	32 (76)	47 (71)	0.57
**Hyperlipidemia (%)**	23 (55)	35 (53)	0.86
**BMI ± SD**	31 ± 6	30 ± 6	0.68
**Family history of CAD (%)**	12 (29)	12 (18)	0.21
**Current smoker (%)**	13 (31)	8 (12)	0.01
**10 yr framingham risk ± SD**	29 ± 19	18 ± 16	0.001

BMI = body/mass index; CAD = coronary artery disease; SD = standard deviation. MPR was calculated as peak CS flow/pre CS flow.

## Discussion

To our knowledge, this is the first study to show that MPR can be rapidly measured using CS flows in the vast majority of patients during regadenoson stress CMR. Acquisition of CS images added approximately 2–3 minutes to overall scanning time with an additional 5 minutes required for off-line quantitative flow analysis and is therefore practical to perform in the clinical setting. Using this method, we have demonstrated that MPR is significantly underestimated when calculated as peak CS flow/post CS flow as compared to peak CS flow/pre CS flow. This difference is abolished when aminophylline is administered immediately after peak CS flow measurement is completed. We have shown that this is because in the absence of aminophylline, residual hyperemia is still present even 15 minutes after regadenoson administration, at the time of resting perfusion acquisition (Figures 6 and 7).

### Comparison with prior studies

A number of prior studies have calculated MPR from phase-contrast CS flow measurements in man and demonstrated good agreement with PET [11,23,24]. However, these were very small studies (n = 9, n = 16 and n = 24) performed in normal volunteers. Koskenvuo et al. measured MPR with dipyridamole using this approach in 20 patients with coronary artery disease and also demonstrated good agreement with PET [18]. Our study is far larger (n = 117) and demonstrates the feasibility of this approach during clinical CMR stress in symptomatic patients presenting with symptoms of possible myocardial ischemia. Our study population is typical of the type of patients presenting to a stress CMR service. This method of measuring MPR is considerably simpler and less labor intensive than traditional quantitative CMR perfusion techniques such as time-signal intensity analyses. In contrast to prior studies we have used the relatively new A2A receptor agonist – regadenoson – which is now the most widely used vasodilator stress agent in the United States [1].

A major finding of our study is that the hyperemic effect of regadenoson persists even after 15 minutes post infusion. Similar results were reported in a recent study by Bhave et al., in which MPR measurements were made in a sample of 20 healthy volunteers with semi-quantitative perfusion analysis using the ratio of maximal myocardial upslopes in the mid-ventricular slice [4]. However, in their study aminophylline did not completely reverse the residual hyperemia induced by regadenoson. Potential explanations for this discrepancy includes: 1) the much smaller sample size used in that study compared to ours (n = 20 vs. n = 117) which increases the possibility of false-positive findings. 2) Differences between the methods used for myocardial blood flow measurement, for example they measured myocardial up-slopes only in the mid-ventricular slice and no measurements were made of the basal or apical regions. In contrast blood flow in CS accounts for the almost all of myocardial blood flow. 3) Their study included only healthy volunteers, which may differ from our population of patients with known or suspected coronary artery disease being evaluated for symptoms of possible myocardial ischemia.

### Clinical implications

#### MPR measurement

The epicardial coronary arteries represent only a tiny fraction of the overall coronary circulation - which extends from the large epicardial conduit arteries through pre-arterioles, arterioles and capillaries before eventually draining back through the venous system into the coronary sinus [25]. MPR depends not only on trans-stenotic pressure gradient of the epicardial arteries and thus stenosis severity but even more on the ability of the coronary microvasculature (especially the pre-arterioles) to dilate [25]. Therefore, coronary microvascular dysfunction, which impairs pre-arteriolar function, reduces MPR independently of the presence of epicardial coronary stenosis [25]. Table 4 shows baseline patient characteristics stratified by MPR < 2 vs MPR ≥ 2. Patients with impaired MPR (<2) were significantly more likely to have advanced age, diabetes, higher Framingham risk score, and history of current smoking. These findings are consistent with the known effects of these risk factors on the coronary microvascular function [25]. Thus the ability to quantitatively measure MPR may allow a more comprehensive understanding of chest pain syndromes and, in particular, of microvascular dysfunction. Indeed, several recent studies using PET have suggested that measurement of MPR provides significant additive prognostic information during stress perfusion imaging in patients with known or suspected CAD [13-16]. However measurement of MPR with both PET and current CMR techniques are cumbersome, and in the case of PET require radiation as well as on-site Rubidium-82 generators. Therefore, they are challenging for routine clinical practice and have been limited to specialized research centers. In this study we have shown that MPR can be rapidly and simply measured using phase-contrast imaging of CS flow during regadenoson stress CMR. We have demonstrated that this is feasible in the large majority of typical patients referred for this procedure. Recent trials have shown the high diagnostic accuracy of stress CMR for the detection of epicardial coronary artery disease [26]. The addition of a simple, rapid method of MPR measurement may provide an additional tool in the CMR armamentarium for evaluating coronary microvascular physiology and pathology which maybe of importance in a number of conditions including coronary artery disease, Syndrome X and hypertrophic cardiomyopathy. Future studies are needed to determine whether this measurement of MPR during regadenoson stress perfusion can provide additive prognostic information as has been demonstrated by PET [13-16].

#### Role of aminophylline

The pharmacokinetics of regadenoson are complex, with an initial phase half-life of approximately 2–4 minutes, followed by an intermediate and late phase, with the latter having a half-life of 2 hours [3,9]. Indeed, we have shown that myocardial blood flow remains elevated despite waiting at least 15 min. Thus in order to avoid underestimating MPR, we would recommend measuring MPR as peak CS flow/pre CS flow rather than peak CS flow/post CS flow. Alternatively either measurement can be used if aminophylline is administered since it reverses the residual hyperemic effect of regadenoson.

The implications of this persistent hyperemia extend beyond the accurate calculation of MPR. If post CS flow does not truly represent pre CS flow (i.e. basal myocardial blood flow), the diagnostic accuracy of the test to detect ischemia may be affected. A region of hypoperfusion that is present at both stress and rest is typically interpreted as artifact [21]. However, if there is persistent hyperemia during the “resting” perfusion images then this region may actually represent ischemia. One potential solution for this would be to perform rest perfusion images prior to stress images ensuring that rest perfusion is truly performed under basal flow conditions. The drawback of this protocol is the risk that gadolinium contrast may accumulate in infarcted areas and potentially mask inducible ischemic regions in subsequent stress perfusion images [27]. An additional advantage of a stress followed by rest protocol, is that if stress perfusion images are completely normal and free from artifact, the rest perfusion images can sometimes be omitted resulting in a faster protocol. An alternate solution would be to reverse the vasodilatory effect of regadenoson immediately after acquisition of stress perfusion images in every case. Our data show that myocardial blood flow does, indeed, return to basal conditions after aminophylline injection. Thus, *routine* administration of aminophylline after vasodilator stress will allow for “clean” resting perfusion images that represent basal flow conditions.

### Limitations

There are a number of important limitations to our study that must be borne in mind. The normal adult CS diameter has been reported as being around 8.3 ± 2.5 mm at mid-diastole [28]. With the current in-plane resolution of 1.3 × 1.3 mm only a limited number of voxels will be located within the lumen of the vessel resulting in partial volume errors. Moreover, the CS is a mobile structure resulting in errors from through and in-plane motion. Misalignment of the imaging plane is another potential source of error. Another major cause of concern is phase-offset errors - particularly given the low velocities in the CS [29]. We did not use phantoms or stationary correction schemes for this study partly to keep scanning and post-processing times within the limits of a typical clinical protocol. There is also considerable anatomic variation in cardiac venous anatomy. In particular, the insertion of the medial cardiac vein maybe very close to the orifice of the CS and thus its contribution to flow volume maybe missed. One might postulate that some of these sources of error may primarily impact absolute blood flow measurements rather than MPR, since presumably similar errors occur under both stress and rest conditions. In this study we did not perform a direct head-to-head comparison of MPR derived from CS flow compared to a standard such as PET since good agreement has previously been reported [11,18,23,24]. Moreover, the association of impaired MPR (<2) in our study with advanced age, diabetes, current smoking and higher Framingham risk score is consistent with the known effects of these variables on coronary microvascular function (Table 4). In this study we did not use other doses or timings of aminophylline administration. However we have shown that 100 mg of aminophylline given in the protocol described reverses the residual hyperemia which would otherwise be present. Although patients were asked to withhold caffeine for at least 12 hours as recommended in the regadenoson package insert, caffeine levels were not drawn to assess for compliance. In this study, patients were referred by their cardiologists for clinically indicated scans. It is likely that our referring cardiologists were aware of CMR contraindications leading to some degree of referral bias since we saw no patients with active bronchospasm, high-grade AV block, metallic implants incompatible with CMR, or significant renal failure during the study period. ECG monitoring in the CMR environment remains challenging and is a limitation of stress-CMR. Finally, although we were able to obtain MPR measurement in the vast majority of patients (108 out of 117 i.e. 92%), there were a few cases where patients were unable to complete the protocol or image quality was too poor for this to be possible.

## Conclusions

We have shown that MPR calculation from CS flow measurements can be successfully performed in the vast majority of symptomatic patients with known or suspected coronary artery disease. Measurement of MPR was relatively rapid and is practical to perform in the clinical setting. Further studies are needed to determine whether this measure of MPR during regadenoson stress perfusion can provide additive prognostic or diagnostic information. We found that residual hyperemia is still present even 15 minutes after regadenoson administration, at the time of resting perfusion acquisition. However, this can be completely reversed by aminophylline. Therefore in a stress-rest CMR protocol, MPR should be calculated as peak CS flow/pre CS flow – particularly if aminophylline is not given. Moreover, our findings imply that it may be preferable to *routinely* give aminophylline when performing stress-rest CMR perfusion using regadenoson, in order to avoid possible misinterpretation of perfusion defects from lingering hyperemia on the “resting” perfusion scans.

## Abbreviations

CAD: Coronary artery disease; CMR: Cardiovascular magnetic resonance; CS: Coronary sinus; LGE: Late gadolinium enhancement; MPR: Myocardial perfusion reserve; PET: Positron emission tomography; SPECT: Single-photon emission computed tomography.

## Competing interests

The authors declare that they have no competing interests.

## Authors’ contributions

AF conceived the study, participated in its design and coordination, supervised imaging and helped to draft the manuscript. VKD participated in the study design and conduct, and drafted the manuscript. MAB and AWE participated in the study coordination and supervised the performance of the studies. CD and RCG monitored the imaging studies. All authors read and approved the final manuscript.
